# Dynamic Impact Characteristics of Airdrop Cushioning Materials and a *C* − *σ*_m_ Curve-Based Cushioning Pad Design Method

**DOI:** 10.3390/ma19122526

**Published:** 2026-06-11

**Authors:** Zhongda Wu, Zhaojun Xi, Yichao Li, Xuesong Xiang, Renfu Li

**Affiliations:** School of Aerospace Engineering, Huazhong University of Science and Technology, Wuhan 430074, China; d202280635@hust.edu.cn (Z.W.);

**Keywords:** airdrop, material cushioning, numerical simulation, optimal stress, lightweight design

## Abstract

To mitigate impact damage to airdropped supplies during landing, this study proposes a cushioning pad design method based on the *C* − *σm* (cushioning coefficient–maximum stress) curve, aiming to balance energy absorption efficiency with lightweight requirements. A medium-sized airdrop impact simulation model is established and validated via drop impact tests, and systematic dynamic impact analyses are performed on three representative cushioning materials: honeycomb paperboard, polyurethane foam, and aluminum foam. Their cushioning characteristic curves are compared, revealing that all three materials exhibit a concave *C* − *σm* profile (first decreasing, then increasing) with distinct optimal stress ranges for airdrop cushioning applications: aluminum foam for high stress (≥500 kPa), polyurethane foam for medium stress (350–450 kPa), and honeycomb paperboard for low stress (≤200 kPa). The energy absorption potential decreases with the optimal stress threshold, while cushion thickness positively correlates with the airdrop load range. In the low-stress stage, the maximum stress shows a strong functional dependence on energy density, rendering thickness effects negligible for energy absorption. Under the material fragility constraint, the *C* − *σm* curve-based graphical method can accurately determine the cushion pad’s optimal thickness and bearing area. In design Case 3, optimizing the bearing area reduced the required cushion thickness from 100.5 cm to 25.0 cm, substantially decreasing the cushion volume. The findings provide reliable material-level insights and theoretical support for impact protection design in airdrop cargo, with clear guidance on selecting cushioning materials based on their intrinsic mechanical response.

## 1. Introduction

Airdrops are critical for emergency response, logistics, and the transportation of special materials; the effectiveness of impact protection during airdrops directly affects the success rate of the operation and the integrity of the cargo [1,2]. Currently, heavy-load airdrops commonly employ airbag cushioning systems mounted beneath the platform to withstand high-energy impacts [3]; however, airbag cushioning is costly and prone to issues such as tipping and overturning. Particularly in airdrop scenarios involving high-altitude regions, complex weather conditions, or lightweight cargo, the effectiveness of airbag cushioning is even more unstable [4]. Consequently, traditional protection methods relying on airbag cushioning struggle to meet the demand for more precise control over the impact acceleration of airdropped items [5]. For the airdrop of small and medium-sized items with relatively low mass, researchers have begun to use cushioning materials to absorb landing impact energy. However, systematic research on the application of different materials in airdrop cushioning remains insufficient. There is an urgent need to thoroughly investigate the energy absorption characteristics of various materials to enhance protection against landing impacts.

Currently used cushioning and energy-absorbing materials primarily include honeycomb paperboard [6], polymer foams (such as polyurethane) [7], and aluminum-based porous materials (such as aluminum honeycomb or aluminum foam) [8]. The dynamic compression behavior of these materials has been extensively studied, and their stress–strain responses under impact loads exhibit strain rate sensitivity and plateau regions. However, regarding the matching relationship between the weight of the dropped object and the brittleness of the cargo, there remains a lack of systematic theoretical methods for selecting appropriate cushioning materials and determining the thickness and bearing area of cushioning pads based on material constitutive properties. Engineering practice currently relies primarily on extensive impact testing for empirical selection.

In low-energy impact scenarios (e.g., electronic product packaging), cushion pad parameters are typically designed based on material dynamic cushioning curves; however, the traditional test methods for plotting these curves are time-consuming and require a large number of samples. To address this, researchers have explored various alternative methods: Burgess [9] proposed a method for constructing cushioning curves based on stress–energy relationships, using a single cushioning curve obtained from measurements at a specific drop height (*h*) and cushion thickness (*t*) to derive cushioning curves for different h/t ratios. Sek et al. [10] introduced a dynamic factor, utilizing the material’s quasi-static compression characteristics and limited dynamic test data to predict cushioning curves. Li et al. [11] further refined the functional form of the dynamic factor to better account for the variation in material compression behavior with the strain rate. Ge [12] provided a systematic review of these methods and recommended that packaging engineers design cushioning solutions based on cushioning coefficient–energy density curves. However, medium-sized airdrops—defined here as those with a mass of less than 1000 kg and an impact velocity of 6–8 m/s—constitute high-load, high-impact-energy scenarios. Their energy density is 1–2 orders of magnitude higher than typical industrial packaging parameters (mass ≤ 50 kg, velocity ≤ 3 m/s). Consequently, cushioning curves generated based on energy density carry a risk of misalignment and are difficult to apply directly to cushioning designs for airdrop impacts.

In the field of drop impact response research, numerical simulation techniques have been widely applied in engineering. Tools such as ABAQUS [13] and LS-DYNA [14,15] can effectively simulate the dynamic behavior of materials under impact loads, with the discrepancy between simulation results and classical cushioning design methods falling within acceptable limits [16,17]. Yau [18] reviewed drop impact analyses conducted on consumer electronic devices such as mobile phones and two-way radios from 2000 to 2010, and discussed the current status of the finite element method in predicting the drop impact performance of electronic devices. Wang et al. [19] performed numerical simulations of drop tests using the finite element method (FEM), focusing on the impact of packaging modeling methods and material model parameter control on simulation accuracy. Muthuram and Saravanan [20] analyzed the dynamic response of PCB boards and the optimization of their supporting structures. However, existing simulation studies mostly focus on local structural optimization or the drop response of small electronic products. There is a lack of a drop impact simulation framework tailored to airdrop systems, and most studies lack reliable experimental data to validate material models and boundary conditions. This severely limits the engineering application of numerical simulation methods in the design of airdrop shock-resistant systems.

To address these shortcomings, this study establishes a simulation model for airdrop impact and systematically investigates the influence of three representative cushioning materials on the impact protection performance of airdropped items: aluminum foam, polyurethane foam, and honeycomb paperboard. These materials were deliberately chosen to cover three distinct categories of lightweight cushioning media with fundamentally different microstructures and mechanical responses, and their optimal stress ranges span high-, medium-, and low-stress domains, respectively. This selection allows for the establishment of a comprehensive material selection framework for a wide range of airdrop loading scenarios. By obtaining the peak accelerations of airdrop cargo under each cushioning pad, the *C* − *σm* curves for the three materials are plotted. Based on this, a quantitative design method for cushioning solutions using the *C* − *σm* curve was developed. By combining this method with typical case studies, the optimal thickness and bearing area of the cushioning pads were determined, enabling a quantitative enhancement design for airdrop cushioning. This method not only addresses the challenges associated with selecting cushioning materials and calculating parameters for airdrop applications, but also offers excellent scalability, making it applicable to other cushioning materials and operating conditions. It thus provides a new approach to research on cushioning protection for airdrop packaging.

## 2. Airdrop Cushioning Packaging

### 2.1. Airdrop Packaging

**Figure 1:**
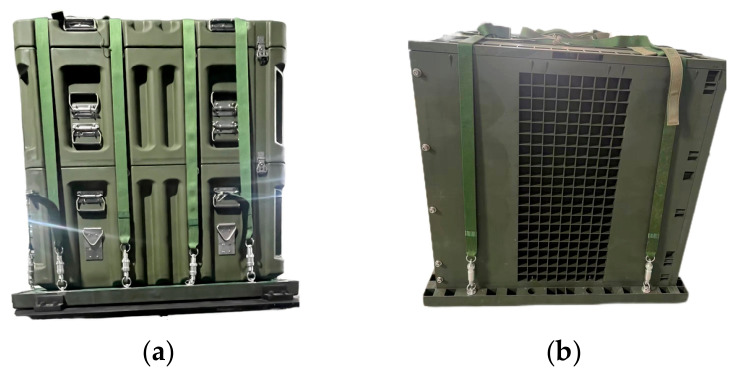
Airdrop cushioning packaging for middle parts: (**a**) equipment box; (**b**) container.

Figure 1 shows the structural configuration of a traditional medium-sized airdrop system and its actual assembly [21]. As shown in Figure 1a, the system primarily comprises core components such as the airdrop platform, cargo containers, restraint straps, guide pulleys, and lashing rings. The equipment boxes are typically secured in a two-layer-stacked configuration via straps onto the platform and are connected to the airdrop parachute pack. Figure 1b presents a photograph of the fully assembled airdrop container. According to current airdrop operational standards, deliverable items are directly strapped and fixed onto the surface of the airdrop platform, which itself lacks any integrated cushioning design. Consequently, upon landing, the impact load is transmitted to the items with minimal attenuation. To ensure the item’s safety, it is urgently necessary to place appropriate cushioning materials between the platform and the item to reduce the landing impact load and enhance the safety of airdropped items. This description of the actual airdrop packaging configuration provides a clear background for the subsequent simulation modeling in this study.

### 2.2. Analysis of Airdrop Impact Process and Packaging

**Figure 2:**
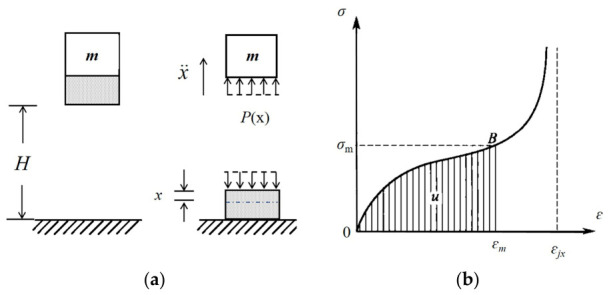
Principle of energy conversion in drop shock process: (**a**) potential gravity of the airdrop; (**b**) elastic potential energy of the cushion pad.

The tray-mounted payloads fall from a height *H*, as shown in Figure 2. At the beginning of the fall, the kinetic energy of the airdropped item is zero, and the gravitational potential energy is *WH*. The gravitational potential energy per unit volume (*u*_1_) of the cushion is

(1) $$u_{1}=\frac{WH}{Ah}=\frac{\sigma_{s}H}{h},$$

Among them, W = *mg*, where *m* represents the mass of the tray-mounted payloads; *A* represents the surface area of the cushion pad (the shadow square in Figure 2a); and *h* represents its thickness. Meanwhile, this equation can be written as $u_{1}=\sigma_{s}H/h$, where *σ*_s_ represents the static stress.

According to the principle of function, the elastic deformation energy of the cushion pad is equal to the product’s impact on the work done by the cushion (Figure 2a). The cushion pad unit volume of the elastic deformation energy is called the elastic specific energy (or energy density), expressed in *u*; that is,

(2) $$u=\int_{0}^{x_{m}}\frac{P}{A}d\left(\frac{x}{h}\right),$$

where *x*_m_ represents the maximum deformation of the cushion pad, *P* represents its impact force, and *P*/*A* = *σ* is the stress. *x*/*h* = *ɛ* is the strain.

When *x* = *x*_m_, *σ* = *σ*_m_, where *σ*_m_ is the maximum stress of the cushion pad and *x*_m_ is the maximum strain of it. Thus, the above equation can be rewritten as

(3) $$u=\int_{0}^{x_{m}}\sigma d\left(\varepsilon \right),$$

According to energy conservation, at the instant of maximum compression, both the kinetic energy and the gravitational potential energy of the payload become zero, and the initial mechanical energy of the payload is completely converted into strain energy absorbed by the cushioning pad. Per unit volume of the cushion, this energy balance gives $u=WH/Ah=\int_{0}^{x_{m}}\sigma d\left(\varepsilon \right)$, where ‘*u*’ is the energy density of the cushioning material—equal to the area under the stress–strain (*σ* − *ε*) curve (Figure 2b).

In the design of engineering cushions, it is necessary to introduce a characteristic parameter that comprehensively characterizes the energy absorption efficiency of the cushioning material. This parameter relates the energy absorption capacity per unit volume (*u*) to the maximum stress (*σ*_m_) generated during the energy absorption process, thereby evaluating the material’s balance between ‘energy absorption’ and ‘stress generation’. This parameter is known as the cushioning coefficient *C*, and its expression is as follows:

(4) $$C=\frac{\sigma_{m}}{u},$$

According to Newton’s second law, ignoring the self-weight of the cushion and the maximum impact force ($P_{m}=m{\ddot{x}}_{m}$) exerted by the cushion pad on the tray-mounted payloads, the peak acceleration of it is

(5) $$G_{m}=\frac{P_{m}}{W}=\frac{A\sigma_{m}}{W}=\frac{\sigma_{m}}{\sigma_{s}}$$

Substituting Equations (4) and (5) into Equation (6), we obtain

(6) $$G_{m}=\frac{\sigma_{m}H}{uh}=\frac{CH}{h}$$

### 2.3. Cushioning Curves and Calculation of Cushioning Pad Thickness

The *C* − *σ*_m_ curve (cushioning coefficient–maximum stress curve) is a core tool for translating a material’s dynamic constitutive relationship into an engineering design curve. These curves are plotted based on the results of laboratory dynamic drop tests. A heavy hammer is dropped from a specified height H and impacts a specimen on a test bench. The elastic specific energy when the specimen’s deformation reaches its maximum value *x*_max_ is denoted as *u*. An accelerometer attached to the specimen measures the maximum acceleration *G*_m_ during the impact process. Therefore, using Equation (6), the maximum stress (*σ*_m_) of the specimen can be calculated as

(7) $$\sigma_{m}=\frac{mG_{m}}{A}$$

Therefore, using the known parameters, another expression for the cushioning coefficient (*C*) is

(8) $$C=\frac{G_{m}h}{H},$$

Therefore, a single drop impact test can determine a single point on the *C* − *σm* curve. By changing the weight of the test specimen and dropping it from the same height, the maximum acceleration measured in the test will also vary. Then, using Equations (7) and (8), the maximum stress *σ*_m_ of the specimen and the corresponding cushioning coefficient *C* are calculated for each weight. Connecting these points forms the material’s dynamic *C* − *σm* curve.

For the airdrop scenarios considered in this study, the descent velocity ranges from 6 m/s to 8 m/s (corresponding to a drop height of 1.84 m to 3.27 m), and the mass of the airdropped items generally does not exceed 1000 kg. Therefore, the proposed methods and conclusions are valid only within the scope of this study. Prior to this study, no suitable cushioning characteristic curves were available for reference regarding the cushioning design of airdrop packaging within this specific mass range. To address this issue, the peak acceleration of the airdropped item is obtained through airdrop simulation to plot a cushioning characteristic curve suitable for airdrop applications, thereby determining the appropriate cushioning materials and thicknesses for different airdrop masses and drop speeds.

The standard dimensions for a general-purpose medium-sized airdrop pallet are 1200 mm × 1200 mm × 110 mm. For cushioning the packaging of airdrop cargo, given the cargo mass m, maximum impact acceleration *G*_m_, and drop height *H*, the contact area (*A*) between the airdrop pallet and the cargo is determined to calculate the required cushioning material thickness (*h*). The calculation formula is as follows:

(9) $$h=\frac{CH}{G_{m}},$$

## 3. Airdrop Simulation Model

### 3.1. Geometric Models and Material Parameters

**Figure 3:**
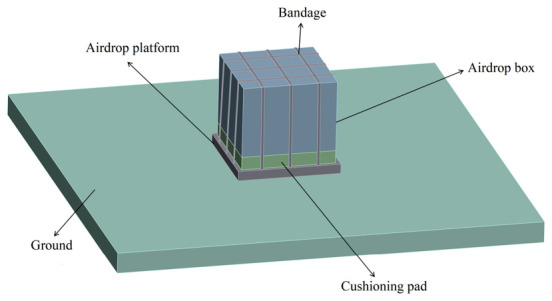
Airdrop assembly model.

The medium-sized airdrop cushioning packaging model for simulation analysis is illustrated in Figure 3. The size of the airdrop tray is known. A large airdrop box is placed on the tray’s cushion pad and secured to the tray via 3 × 3 transverse and longitudinal binding straps. To accurately simulate actual impact dynamics during airdrop landing, precise material mechanical parameters were assigned for the airdrop box, ground, tray, and cushion materials. The airdrop box is fabricated from high-strength ABS plastic, assumed to contain uniformly distributed cargo, with its center of gravity at the geometric center. The total airdrop mass is adjusted by varying the box’s density. The tray is constructed from high-strength nylon 66, while six polyester straps securely connect the box to the tray. The ground is modeled as high-strength concrete. These material parameters are presented in Table 1.

### 3.2. Cushioning Pad Parameters

#### 3.2.1. Honeycomb Paperboard

**Figure 4:**
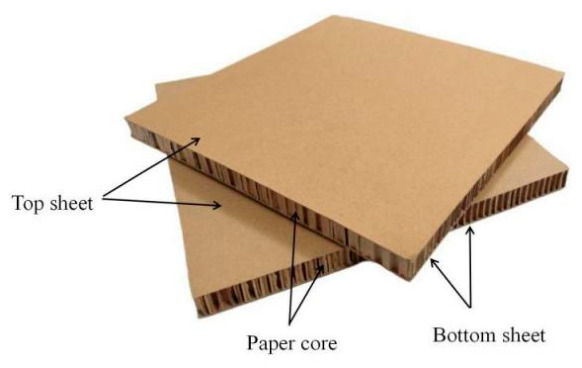
Honeycomb paper specimen.

**Figure 5:**
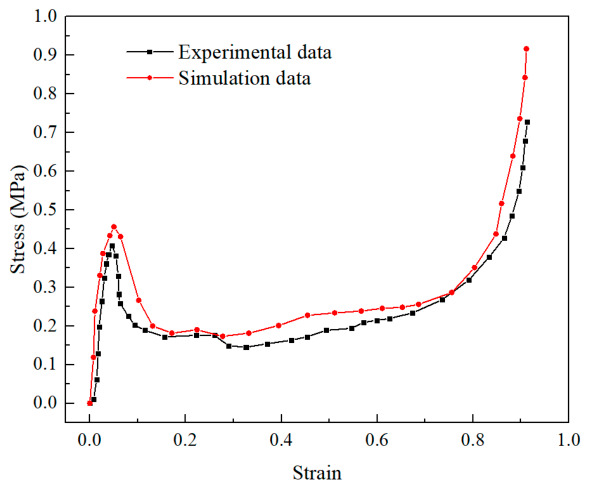
Experimental and simulation comparison of stress–strain curves.

The cushion pads in this study employ three materials: honeycomb paperboard, rigid polyurethane foam, and aluminum foam. Among them, the honeycomb paperboard features a Type A honeycomb core with an incircle diameter of 10 mm. The specific mechanical parameters of the honeycomb paperboard during compression are closely related to its compressive strain rate. Given that the landing velocity of the airdrop platform generally does not exceed 6–8 m/s and the honeycomb paperboard thickness is 100 mm, the average strain rate can be estimated to be no more than 80/s. Dynamic compression tests were conducted on a drop hammer testing machine, with specimen planar dimensions of 100 mm × 100 mm. The honeycomb paper specimens used in the tests are shown in Figure 4. The dynamic compression test results of the same honeycomb paperboard at a velocity of 6 m/s are presented in Figure 5. It can be observed that the compression process of the honeycomb paperboard under external force is divided into four stages: elastic, yielding, plateau, and densification.

For material modeling, a bilinear isotropic hardening model was adopted. In this study, we used the PIECEWISE_LINEAR_PLASTICITY model, which describes strain rate-dependent material behavior [24]. The honeycomb paperboard parameters are listed in Table 2. The cushioning performance of honeycomb paperboard in airdrop applications primarily depends on its out-of-plane compression behavior. During this process, the progressive plastic buckling and crushing of the honeycomb walls generate a stable plateau stress over a wide range of strains. This energy absorption mechanism is primarily determined by the plateau stress and densification strain, rather than the relatively low in-plane Young’s modulus. Therefore, provided that the thickness and bearing area are properly designed, honeycomb paperboard can provide effective impact protection even with relatively low elastic stiffness. We established a numerical model matching the experimental setup and applied identical impact conditions to the honeycomb paperboard model. A comparison between experimental and simulated stress–strain curves (Figure 5) indicates that simulation results closely match experimental data, with consistent response processes.

#### 3.2.2. Polyurethane Foam and Aluminum Foam

The basic mechanical properties of the polyurethane foam and aluminum foam materials selected for this study are listed in Table 1. The porosity of the aluminum foam is 80.8%. Since the landing cushioning method during an airdrop involves a one-dimensional compression process to some extent, both materials were simulated using the MAT_63 (MAT_CRUSHABLE_FOAM) crushable foam material model in LS-DYNA R15.0. This model is based on the one-dimensional crushing assumption and is widely used in the numerical simulation of the dynamic mechanical behavior of foam materials [25]. In the material parameter settings, the stress–strain curves for the two foam materials were discretized into multiple data points and entered into the simulation model as load curves (as shown in Table 3). At the same time, the model’s default unloading rule was used; the unloading process followed the material’s initial elastic modulus, and no additional hysteresis factors were added.

**Figure 6:**
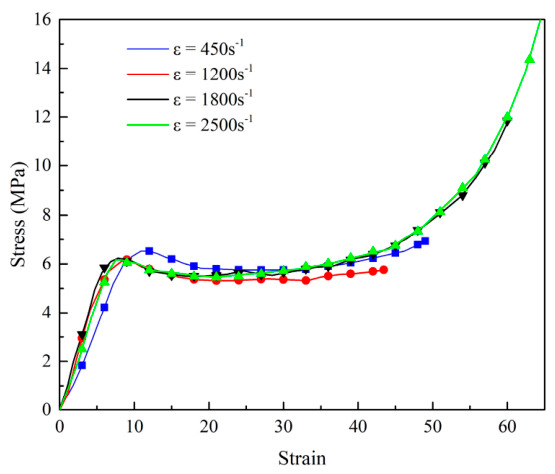
Stress-strain curves of polyurethane foam at different strain rates [24].

To fully investigate the influence of the strain rate (ε) on the two cushioning materials under dynamic impact conditions in this study, targeted verification was conducted by combining dynamic test data from the existing literature with theoretical analysis. Regarding polyurethane foam, the existing published literature [22] has obtained its compression stress–strain characteristic curves within a high-strain-rate range of 450 s^−1^ to 2500 s^−1^ through dynamic impact tests (as shown in Figure 6). The results indicate that the mechanical response of polyurethane foam under high-strain-rate loading conditions varies very little, the stress–strain curves show a high degree of overlap, and the mechanical properties are insensitive to the strain rate. For aluminum foam, the existing literature, [23,26], indicates that, under low-speed impact loading, the dynamic compressive stiffness and plateau stress of foamed aluminum are essentially consistent with static compression results; the strain rate hardening effect is extremely weak, and its influence on the overall cushioning response is negligible. Since the airdrop landing process in this study falls entirely within the low-speed impact range, the stress–strain curves obtained from static compression tests were used to input the model for simulation calculations. This assumption is supported by sufficient literature evidence and is well suited for the operating conditions, capable of meeting the accuracy requirements for dynamic impact simulation [27].

### 3.3. LS-DYNA Model Setup and Meshing

**Figure 7:**
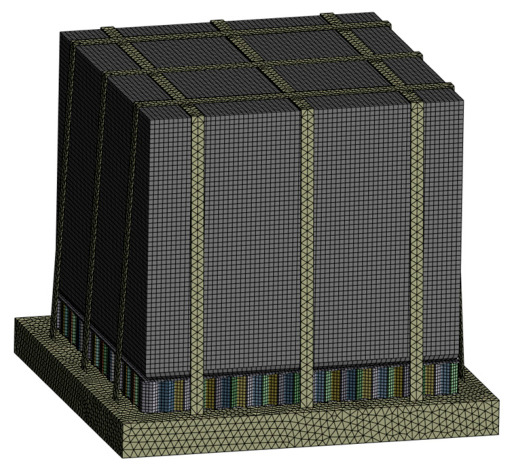
The overall meshing scheme of the airdrop assembly.

This study employed ANSYS/LS-DYNA R15.0 to simulate the airdrop package’s deformation and cushioning response during ground impact. In the model, the airdrop tray and the tiedown straps were treated as a single bound entity. An automatic meshing method is adopted, generating tetrahedral elements with an 18 mm size. The honeycomb paperboard surface layer was meshed with 8-node hexahedral elements, while the core layer was discretized using shell elements (both with 2 mm mesh size). Polyurethane and aluminum foam pads (rectangular blocks) were meshed with regular hexahedral elements (10 mm size). The ground was modeled as a rectangular block discretized with 50 mm hexahedral elements. To verify mesh convergence and ensure simulation accuracy, this study employed three sets of meshes with different densities—coarse, medium, and fine—to conduct mesh-invariant verification. The global characteristic element sizes for the coarse, medium, and fine meshes were set to 1.5, 1.0, and 0.75 times the reference size, respectively. Under identical boundary conditions and loading conditions, a comparative analysis was performed using the peak acceleration during the airdrop impact process as the core evaluation metric, as detailed in Table 4. The results indicate that, using the fine-mesh calculation results as the reference, the relative error of the reference mesh is only 1.5%; the results tend to converge, proving that the current mesh size meets the computational accuracy requirements. The overall mesh model of the honeycomb paperboard airdrop assembly is shown in Figure 7.

The simulation conditions and boundary conditions are based on actual airdrop operations, disregarding the relative motion of items within the container. The initial descent velocity of the airdrop assembly is set to *V*_0_ = 6 m/s, the acceleration due to gravity is *g* = 9.8 m/s^2^, and the ground model is assumed to be completely stationary. Regarding contact relationships, nonlinear solid contact is used between the equipment container, cushioning pads, loading platform, and the ground. The Pure Penalty function is employed to avoid model penetration issues, and all contact pairs are defined according to the master–slave surface rule to ensure computational stability and accuracy. Specifically, the contact between the bottom surface of the airdrop box and the cushion, as well as between the cushion and the ground, uses the AUTOMATIC_ SURFACE_TO_SURFACE algorithm. The static friction coefficient is set to 0.2, and the kinetic friction coefficient to 0.15.

To improve the stability and accuracy of the simulation calculations, this paper establishes a comprehensive set of solution control parameters: to suppress spurious high-frequency oscillations during the simulation, stiffness-weighted Rayleigh damping is applied globally, with damping parameters set to α = 0 and β = 1 × 10^−4^. The simulation time step is automatically calculated based on the minimum element characteristic length of the model and the material sound speed, with a scaling factor of 0.9 applied; the initial time step is approximately 1.2 × 10^−4^ ms, ensuring that the Courant number remains stably below 0.9 throughout the simulation. To eliminate the interference of the hourglass effect on computational accuracy, all solid elements employ the Flanagan–Belytschko stiffness formulation (IHQ = 4) for hourglass control, with the hourglass coefficient set to 0.05.

### 3.4. Test Validation

**Figure 8:**
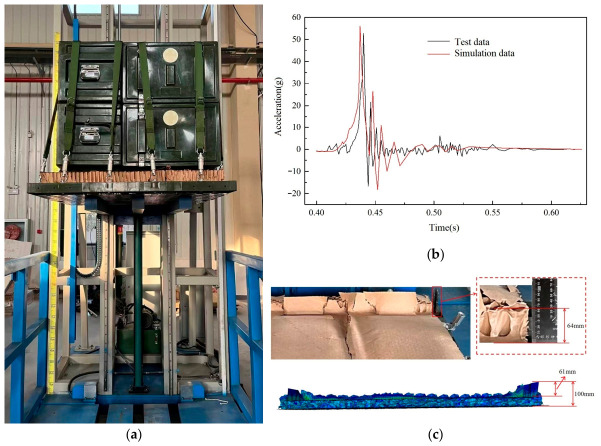
Comparison of drop test and simulation of airdropped assemblies: (**a**) drop test; (**b**) acceleration comparison; (**c**) test and simulation compression comparison.

To validate the accuracy of the simulation results, a drop test was conducted by securing the airdrop box and honeycomb paperboard onto a pallet. During the test, the acceleration of the airdrop box was measured, and the deformation of the honeycomb paperboard after impact was recorded. The drop test setup is shown in Figure 8a. A corresponding simulation model was established based on the test configuration. Using the free-fall formula ($h=v^{2}/2g$), the drop height corresponding to an impact velocity of 6 m/s was calculated as 1.84 m. A zero-height drop tester (model QD-B9255, manufactured by Haida International Instrument Co., Ltd., Dongguan, China) was used to lift the 300 kg airdrop package to this height and release it for the test. An accelerometer was fixed to the box to collect acceleration data.

Figure 8b presents the acceleration–time curves obtained from both the test and the simulation during the box drop impact onto the ground. The results show a high degree of agreement in the overall trend between the simulation and the experimental data. Both curves clearly illustrate the typical process of an impact event: the acceleration rapidly increases from its initial value to a peak, followed by oscillatory decay until it eventually returns to zero. The primary oscillation patterns and decay trends of the curves are also largely consistent. This indicates that the established simulation model accurately captures and reproduces the actual physical process. The peak impact overloads obtained from the test and the simulation were 52.8 g and 54.6 g, respectively, with a difference of 1.8 g and a relative error of 3.4%. Figure 8c compares the simulated and experimental deformation patterns of the honeycomb paperboard and shows general agreement in both the crushing mode and the amount of compression.

To further validate the numerical model under varying weight conditions, drop tests were conducted on airdropped components of different masses. The measured peak accelerations, along with the corresponding simulation values and relative errors, are presented in Table 5. The comparative analysis shows that the simulation results show good agreement with the experimental data, with relative errors below 10% in all but one case. Besides sensor drift and measurement noise, several factors may account for the observed errors. First, there are discrepancies between the simulated geometric model and the actual test setup, including simplifications of the load platform and the use of equivalent material models. Second, the strapping configuration in the simulation was idealized and differs from the actual binding method used in the test. Finally, numerical inaccuracies inherent in the simulation, as well as the time integration accuracy in ANSYS/LS-DYNA R15.0, also influence the results. In conclusion, the simulation demonstrates strong consistency with experimental data in terms of both temporal response (impact acceleration) and spatial deformation (compression of honeycomb paperboard). The accuracy of the drop simulation under various weights is confirmed, with all observed deviations lying within acceptable engineering tolerances. It is important to note that the experimental drop test validation described above was performed exclusively for the honeycomb paperboard cushioning system. The subsequent numerical analyses for polyurethane foam and aluminum foam cushioning systems are based entirely on simulation predictions using the material input curves described in Section 3.2.2. Although simulation predictions were performed using published material data for these two materials, it has not yet been directly validated through physical testing in this study.

## 4. Results and Discussion

### 4.1. Honeycomb Paperboard Cushioning Characteristics and Curves

To investigate the cushioning properties of honeycomb paperboard during airdrop impact, a simulation analysis was conducted for the process of airdrop containers of varying weights striking the ground. Peak acceleration was extracted, and the maximum stress and cushioning coefficient were calculated. The simulation parameters were set as follows: the mass of the airdrop containers started at 25 kg and was gradually increased until the acceleration increase exceeded 50%. The bottom cushioning pad consisted of honeycomb paperboard measuring 1100 mm × 1100 mm × 150 mm, the drop impact velocity was 6 m/s, and the corresponding drop height was 1.84 m. The peak acceleration (*G*_m_) obtained from the simulation results, as well as the static stress (*σ*_s_), maximum stress (*σ*_m_), and cushioning coefficient (*C*) calculated using Equations (1), (7), and (8), are listed in Table 5.

**Figure 9:**
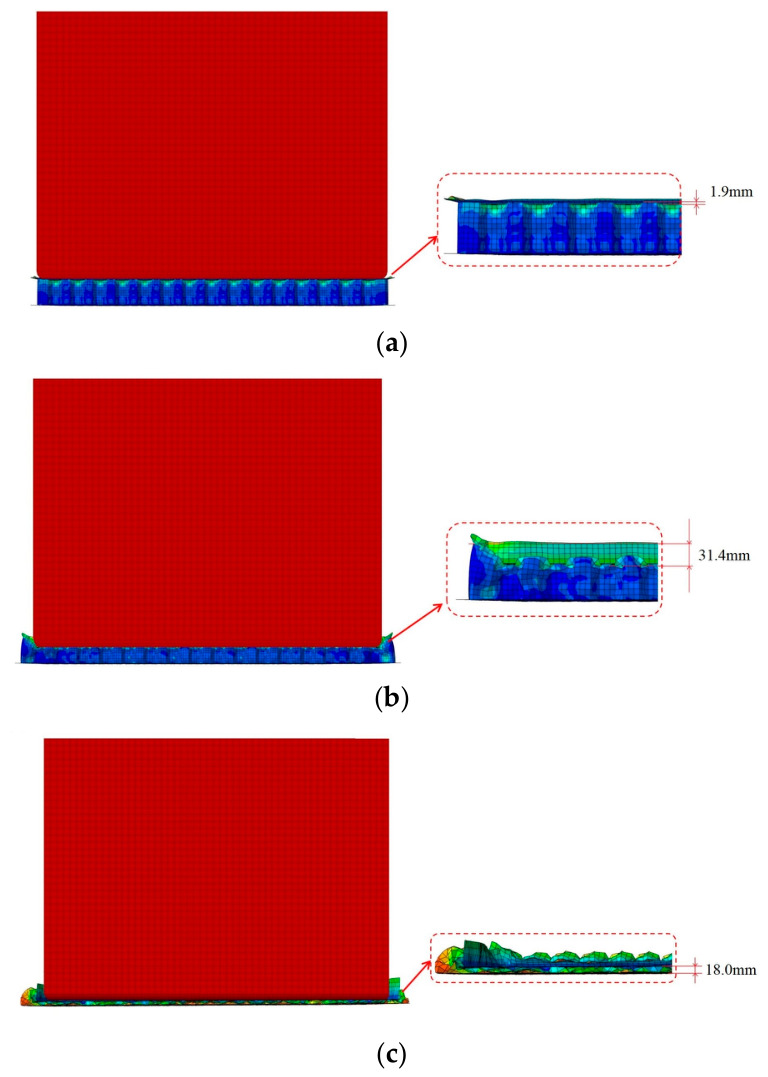
Cushioning performance of honeycomb paperboard under the drop impact of different weights: (**a**) 25 kg; (**b**) 300 kg; (**c**) 600 kg.

As shown in Table 6, the overload value *G*_m_ corresponding to the peak acceleration is significantly higher when the airdrop container has a lighter mass. As the mass gradually increases, the *G*_m_ value exhibits a trend of ‘first decreasing to a minimum, then gradually increasing.’ This phenomenon is closely related to the nonlinear mechanical properties of honeycomb paperboard: for lightweight airdrop containers, at the moment of contact with the honeycomb paperboard, the board undergoes only a brief linear–elastic compression phase. Within an extremely short displacement (compression of 1.9 mm; see Figure 9a), the container is rapidly decelerated to a stop. At this point, the dynamic peak force generated by the honeycomb paperboard far exceeds the plateau stress, resulting in a high *G*_m_ value; For medium-mass airdrop containers with moderate inertia, the cellular structure of the honeycomb paperboard is stably crushed (compression of 31.4 mm, see Figure 9b). The compression time is extended, the peak force is limited to near the plateau stress, and the *G*_m_ value gradually decreases to reach an optimal level. For high-mass airdrop containers, as mass continues to increase, the honeycomb paperboard cells are gradually compacted (18.0 mm remaining, see Figure 9c), energy absorption efficiency decreases, and the peak force rises again until reaching a state of energy absorption saturation.

By combining the method of sudden changes in the overload growth rate with the method of steep increases in maximum stress, a quantitative analysis of the data in Table 5 was conducted to accurately determine the energy absorption saturation point of the 150 mm honeycomb paperboard: using the overload growth rate *k* = Δ*G*_m_/Δm between adjacent load segments as the core criterion, when *k* suddenly increases by ≥50% and the maximum stress simultaneously rises exponentially, this is identified as the critical point of energy absorption saturation [27]. Calculations show that, at 540 kg, *G*_m_ reaches its minimum value (28.75 g), corresponding to a maximum stress of 125.65 kPa; in the 540–580 kg range, the overload growth rate *k* = 0.11; in the 580–600 kg range, the overload growth rate *k* surges to 0.55, while the *σ*_m_ rises from 155.65 kPa to 215.01 kPa, a 38% increase; in the subsequent 600–700 kg range, both the *G*_m_ and *σ*_m_ exhibit explosive growth; at 700 kg, *G*_m_ rises to 66.68 g and the *σ*_m_ reaches 374.56 kPa, indicating that the material has densified. Based on these results, the energy absorption saturation threshold for the 150 mm honeycomb paperboard was determined to be 580 kg, corresponding to a critical maximum stress of 155.65 kPa and a critical overload of 33 g. The effective energy absorption range is below 580 kg; at or above 580 kg, the material cannot sustain energy absorption through plastic deformation, and its cushioning capacity drops sharply.

**Figure 10:**
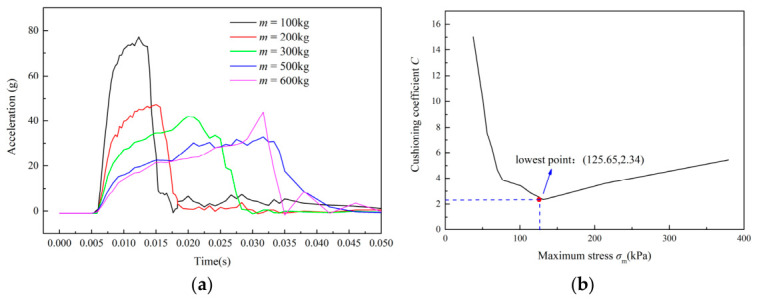
Cushioning performance of honeycomb paperboard: (**a**) *G*_m_-time curve; (**b**) C−σm curve.

Figure 10a shows the peak acceleration (*G*_m_)–time curves during drop tests of airdrop containers with different weights. With the same cushioning material, heavier containers (before saturation) dissipate energy longer and absorb more gravitational potential energy. Their acceleration peaks decrease gradually and the curves become flatter. When the weight exceeds 580 kg (saturation threshold), acceleration peaks rise sharply and curves become steeper, showing typical energy absorption saturation. Figure 10b shows the maximum stress (*σ*_m_) versus the cushioning coefficient (*C*) for different weights. The concave curve has a minimum point, meaning that the material has an optimal stress range for cushioning. When *σ*_m_ reaches 0.155 MPa, the curve becomes more curved, consistent with common cushioning materials. A smaller *C* means a higher energy absorption efficiency. Thus, the optimal design is to achieve the minimum *C* (540 kg, *C* = 2.35). The design load must stay below the saturation threshold to ensure stable cushioning performance.

### 4.2. Cushioning Characteristics of Different Thicknesses and Stress-Energy Method

**Figure 11:**
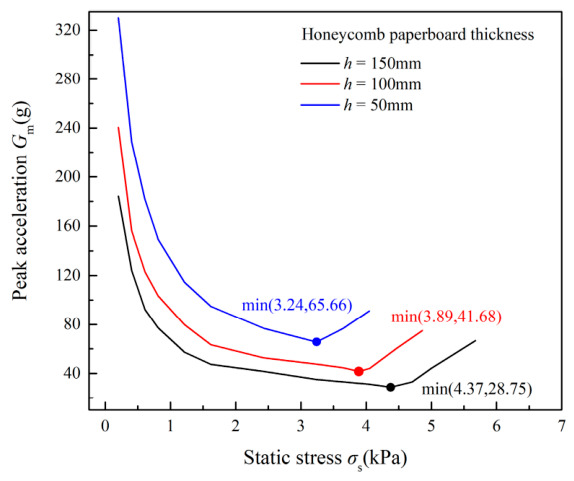
Static stress–peak acceleration (*σ*_s_ − *G*_m_) curves of different thicknesses.

Drop impact simulations were conducted on honeycomb paperboard with thicknesses of 50 mm, 100 mm, and 150 mm under a drop velocity of 6 m/s. A systematic and comprehensive analysis of their cushioning performance was performed using the static stress–peak acceleration (*σ*_s_ − *G*_m_) cushioning curve, the energy density versus maximum stress (*u* − *σ*_m_) curve, and the constitutive fitting relationship. As shown by the *σ*_s_-*G*_m_ cushioning curve (Figure 11), under the same *σ*_s_ (i.e., the same weight of the drop test box), the thicker the honeycomb paperboard, the lower the *G*_m_, and the better the cushioning protection performance. This is essentially because thicker paperboard provides a longer cushioning stroke, enabling it to absorb the impact energy of the drop more effectively. The cushioning curves for all three board thicknesses exhibit a typical U-shaped profile, decreasing initially and then increasing: when the *σ*_s_ is too low, the board is not sufficiently compressed, resulting in an insufficient effective cushioning stroke and low energy absorption efficiency, which leads to a high *G*_m_; when the *σ*_s_ is too high, the board undergoes excessive crushing and compaction, losing its cushioning capacity, causing *G*_m_ to rise again. The minimum value of the curve corresponds to the optimal cushioning conditions for the paperboard. The optimal conditions for 50 mm, 100 mm, and 150 mm thick paperboard are (*σ*_s_ = 3.24 kPa, *G*_m_ = 65.66 g), (*σ*_s_ = 3.89 kPa, *G*_m_ = 41.68 g), and (*σ*_s_ = 4.37 kPa, *G*_m_ = 28.75 g), respectively. As the paperboard thickness increases, the optimal *G*_m_ decreases significantly, while the optimal *σ*_s_ gradually increases. This indicates that thicker paperboard not only offers superior cushioning performance but can also accommodate heavier airdrop payloads.

**Figure 12:**
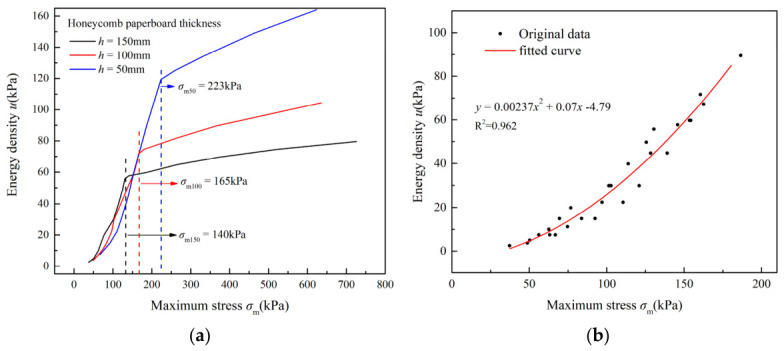
Cushioning curve of different thicknesses: (**a**) *u* − *σ*_m_ curves; (**b**) fitting curve.

Figure 12a shows the *u* − *σ*_m_ curves for honeycomb paperboard cushioning pads of different thicknesses (See Table S1 in the Supplementary for the data). As can be seen from the curves, the relationship between *u* and *σ*_m_ of the honeycomb paperboard exhibits distinct phase-dependent characteristics. In the low-stress stage, the *u* − *σ*_m_ curves for the three board thicknesses closely overlap and exhibit an approximately linear growth trend, indicating that thickness has a negligible effect on the energy absorption characteristics per unit volume of the board at this stage, and that the energy absorption capacity is primarily determined by the stress level. Once *σ*_m_ exceeds a critical inflection point, the curves diverge significantly. At the same *σ*_m_, the thinner the board, the higher the *u*. That is, thin boards exhibit superior energy absorption efficiency per unit volume in the high-stress stage, while the increase in energy density for thick boards is more gradual. This critical inflection point corresponds to the critical stress at which the honeycomb paperboard structure yields and compacts. Thinner paperboard enters the plastic energy absorption-dominated stage more readily, whereas thicker paperboard exhibits greater structural stability and a delayed onset of plastic deformation.

To establish a unified constitutive relationship for the low-stress stage, the experimental data for the three paperboard thicknesses prior to the critical inflection point were fitted using a quadratic polynomial (Figure 12b), yielding the following constitutive relationship between energy density *u* and maximum stress *σ*_m_:

(10) $$u=0.00237\sigma_{m}^{2}+0.07\sigma_{m}-4.79,$$

The coefficient of determination (*R*^2^) for this quadratic polynomial fit is 0.962, indicating excellent fitting accuracy. The results show that, in the low-stress range (*σ*_m_ ≤ 150 kPa), the energy density of honeycomb paperboard follows the same constitutive law as the maximum stress; the effect of thickness on energy absorption characteristics in this range is negligible. This formula can be used to accurately predict the energy absorption performance of honeycomb paperboard under low-stress conditions. In summary, honeycomb paperboard exhibits excellent cushioning performance under low-static-stress (light-load) conditions and is suitable for light-weight airdrop scenarios ranging from 2 to 5 kPa; however, under high-static-stress (heavy-load) conditions, its limitations—such as low plateau stress and insufficient load-bearing capacity—become apparent, making it difficult to meet the airdrop protection requirements for heavy equipment. Therefore, comparative studies on high-load-bearing cushioning materials need to be conducted.

### 4.3. Analysis of Cushioning Characteristics of Different Materials

**Figure 13:**
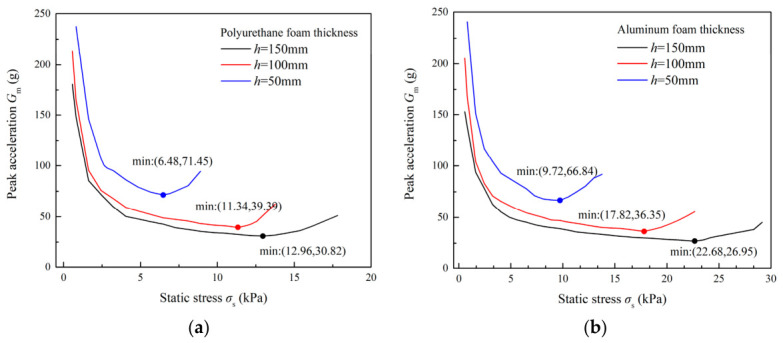
Static stress–peak acceleration curves of cushioning pads with different thicknesses under drop impact: (**a**) polyurethane foam; (**b**) aluminum foam.

To address the issue of insufficient cushioning performance of honeycomb paperboard under high-load airdrop conditions, this study selected aluminum foam and polyurethane foam as alternative cushioning liners. Under consistent boundary conditions—including contact area, thickness, and drop height—drop impact simulations were conducted to obtain the static stress (*σ*_s_)-versus-peak acceleration (*G*_m_) curves (Figure 13) for these two materials at different thicknesses (50 mm, 100 mm, 150 mm), and a systematic comparative analysis was conducted with the experimental results of honeycomb paperboard from previous studies. All three curves exhibit a typical concave (U-shaped) characteristic: as the *σ*_s_ increases, the *G*_m_ first drops rapidly to a minimum point and then gradually rises again. This pattern indicates that all cushioning materials have an optimal static stress condition for cushioning. The underlying physical mechanism is consistent with that of honeycomb paperboard: when the static stress is too low, the material is not fully compressed, resulting in insufficient energy absorption; when the static stress is too high, the material becomes densified, leading to cushioning failure; the minimum value corresponds to the optimal compression state. Optimal cushioning parameters for the two materials are as follows: polyurethane foam: 50 mm (6.48 kPa, 71.45 g), 100 mm (11.34 kPa, 39.39 g), 150 mm (12.96 kPa, 30.82 g); aluminum foam: 50 mm (9.72 kPa, 66.84 g), 100 mm (17.82 kPa, 36.35 g), 150 mm (22.68 kPa, 26.95 g).

Under the same *σ*_s_, the *G*_m_ of both aluminum foam and polyurethane foam decreases significantly as material thickness increases; this pattern is identical to that observed in honeycomb paperboard. The fundamental reason lies in the fact that increased thickness provides a longer compression stroke, allowing the material to more fully dissipate the kinetic energy during the drop, thereby effectively reducing the impact acceleration and enhancing the reliability of the cushioning protection. As material thickness increases, the optimal cushioning static stress *σ*_s_ for all three materials shows an upward trend, but the rate of increase varies significantly, as shown in Table 7. The increase in the optimal load-bearing capacity of aluminum foam and polyurethane foam with increasing thickness is far greater than that of honeycomb paperboard, indicating that these two materials are better suited to accommodate heavy-load airdrop scenarios by increasing thickness. The minimum *G*_m_ for all materials continues to decrease with increasing thickness. Taking a thickness of 150 mm as an example, the minimum *G*_m_ for aluminum foam is 26.95 g; for polyurethane foam it is 30.82 g, and for honeycomb paperboard it is 28.75 g. This indicates that, under their respective optimal conditions, all three materials can achieve excellent cushioning performance, but thicker versions of the materials offer superior overall protective performance.

A comparison with the curve for honeycomb paperboard shows that, in the low-static-stress (light load) range, the *G*_m_ values for aluminum foam and polyurethane foam are significantly higher than those for honeycomb paperboard. This indicates that these two materials possess higher initial stiffness and contact stiffness, and thus exhibit poorer cushioning performance than honeycomb paperboard under light-load airdrop conditions. Therefore, honeycomb paperboard is more suitable for light-load airdrop scenarios within the low-static-stress range (2–5 kPa). Conversely, in the high-static-stress (heavy-load) range, because the plateau stress of aluminum foam and polyurethane foam is far higher than that of honeycomb paperboard, they can absorb more impact energy per unit volume, significantly enhancing their load-bearing capacity. This results in a *G*_m_ value far lower than that of honeycomb paperboard, effectively overcoming the cushioning limitations of honeycomb paperboard under high loads. Based on the cushioning curve data for 150 mm thickness, the three materials form distinct load-matching ranges in airdrop cushioning systems: aluminum foam is suitable for high-static-stress (20–25 kPa) heavy-load airdrop scenarios; polyurethane foam is suitable for medium-static-stress (10–15 kPa) medium-load airdrop scenarios; and honeycomb paperboard is suitable for light-weight airdrop scenarios with low static stress (2–5 kPa). Additionally, as material thickness decreases, the applicable static stress ranges for each of the three materials correspondingly narrow, indicating that thicker cushioning materials have a broader load-adaptive range, whereas thinner materials are better suited for specific, narrower load conditions.

**Figure 14:**
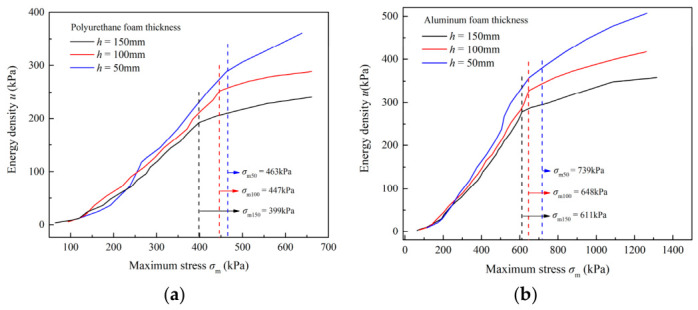
Energy density *u* and maximum stress *σ*_m_ for cushioning pads of different thicknesses: (**a**) polyurethane foam; (**b**) aluminum foam.

To further elucidate the cushioning mechanisms of foamed aluminum and polyurethane foam, a study was conducted on the relationship between energy density *u* and maximum stress *σ*_m_ for cushioning pads of different thicknesses made from these two materials (Figure 14). The results indicate that the *u* − *σ*_m_ relationship for both materials is consistent with that of honeycomb paperboard, exhibiting distinct phase transitions: in the low-stress phase (where *σ*_m_ does not exceed the critical transition point for structural yield/compaction), the energy density u increases approximately linearly with the maximum stress *σ*_m_ (See Tables S2 and S3 in the Supplementary for the data). This phase corresponds to the material’s elastic/quasi-elastic deformation stage, during which structural integrity is maintained and energy absorption is primarily driven by elastic strain energy. Once *σ*_m_ exceeds the critical value, the material undergoes large-scale plastic collapse, stress rises rapidly, and the energy absorption mechanism shifts to be dominated by plastic energy absorption.

**Figure 15:**
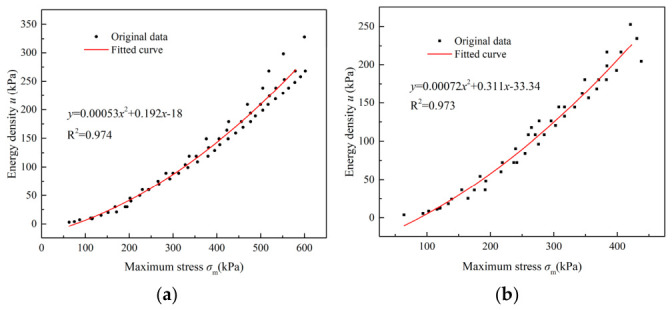
Energy density-maximum stress fitted curves: (**a**) aluminum foam; (**b**) polyurethane foam.

To establish a unified constitutive relationship for the low-stress stage, quadratic polynomial fitting was performed on the experimental data of the three-thickness cushioning pads prior to the stress inflection point. The fitting results are shown in Figure 15a (aluminum foam) and Figure 15b (polyurethane foam). The fitted constitutive relationship for foamed aluminum is

(11) $$u=0.00053\sigma_{m}^{2}+0.192\sigma_{m}-18,$$

The fitted constitutive relationship for polyurethane foam is

(12) $$u=0.00072\sigma_{m}^{2}+0.311\sigma_{m}-33.34,$$

The fitted curves for both materials exhibit an extremely high degree of fit with the original data points, indicating that, during the low-stress (elastic/quasi-elastic) stage, the energy density and maximum stress of aluminum foam and polyurethane foam of different thicknesses follow a unified constitutive law. Thickness has no significant effect on the energy absorption characteristics during this stage. The above equations can be used to accurately predict the energy absorption performance of both materials under low-stress conditions, providing a theoretical basis for the design of airdrop cushioning pads.

### 4.4. Analysis of the Characteristics of the Cushioning Coefficient

**Figure 16:**
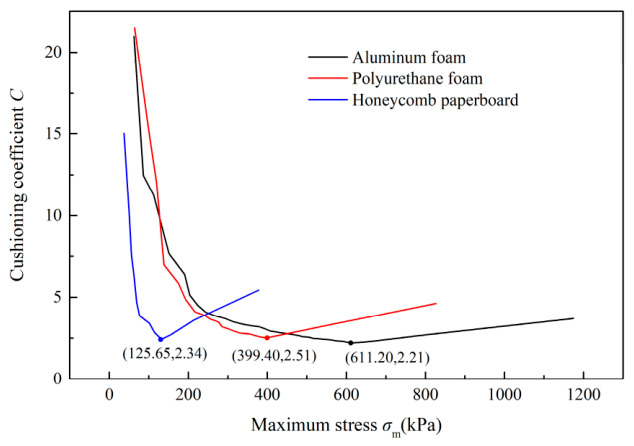
Cushioning coefficient–dynamic stress curves of three cushioning materials.

This section presents a simulation-based comparative analysis based on the *C* − *σm* relationship curves of three cushioning materials, in conjunction with the *σ*_s_ − *G*_m_ cushioning characteristics and *u* − *σ*_m_ energy absorption characteristics discussed earlier. Figure 16 shows the *C* − *σm* relationship curves for three materials: aluminum foam, polyurethane foam, and honeycomb paperboard (See Tables S4–S6 in the Supplementary for the data). The curves for all three materials exhibit a typical U-shaped (concave) pattern: the cushioning coefficient *C* decreases rapidly as the maximum stress *σ*_m_ increases, reaches a minimum, and then gradually rises again. This pattern reflects the evolution mechanism of the energy absorption efficiency of cushioning materials: when *σ*_m_ is low, the material is not fully compressed, the effective energy-absorbing stroke is insufficient, and the energy absorption efficiency per unit volume is low, resulting in a relatively high cushioning coefficient *C*; when *σ*_m_ exceeds a critical value, the material enters the plastic collapse stage, energy absorption efficiency continues to increase, and the *C* value drops rapidly. The minimum point on the curve corresponds to the operating condition with optimal cushioning efficiency. At this point, the material fully utilizes the compression stroke to achieve high-efficiency energy absorption without entering the densification stage; the stress per unit of absorbed energy is minimal, and cushioning performance is optimal. When *σ*_m_ further increases beyond the optimal value, the material undergoes excessive densification, causing stress to rise rapidly while energy absorption efficiency slows, leading to a gradual recovery of the *C* value and a deterioration in cushioning performance.

This section presents a simulation-based comparative analysis based on the *C* − *σm* relationship curves of three cushioning materials, in conjunction with the *σ*_s_ − *G*_m_ cushioning characteristics and *u* − *σ*_m_ energy absorption characteristics discussed earlier. Figure 16 shows the *C* − *σm* relationship curves for three materials: aluminum foam, polyurethane foam, and honeycomb paperboard (See Tables S4–S6 in the Supplementary for the data). The curves for all three materials exhibit a typical U-shaped (concave) pattern: the cushioning coefficient *C* decreases rapidly as the maximum stress *σ*_m_ increases, reaches a minimum, and then gradually rises again. This pattern reflects the evolution mechanism of the energy absorption efficiency of cushioning materials: when *σ*_m_ is low, the material is not fully compressed, the effective energy-absorbing stroke is insufficient, and the energy absorption efficiency per unit volume is low, resulting in a relatively high cushioning coefficient *C*; when *σ*_m_ exceeds a critical value, the material enters the plastic collapse stage, energy absorption efficiency continues to increase, and the *C* value drops rapidly. The minimum point on the curve corresponds to the operating condition with optimal cushioning efficiency. At this point, the material fully utilizes the compression stroke to achieve high-efficiency energy absorption without entering the densification stage; the stress per unit of absorbed energy is minimal, and cushioning performance is optimal. When *σ*_m_ further increases beyond the optimal value, the material undergoes excessive densification, causing stress to rise rapidly while energy absorption efficiency slows, leading to a gradual recovery of the *C* value and a deterioration in cushioning performance.

Table 8 shows a comparison of the cushioning characteristics of the three materials. The lowest point on the *C* − *σm* curve for foamed aluminum corresponds to the coordinates (*σ*_m_ = 611.20 kPa, *C* = 2.21). Among the three materials, it exhibits the highest maximum stress and the lowest C value at the point of optimal cushioning efficiency. Beyond this minimum point, the slope of the *C* − *σm* curve for foam aluminum is extremely gentle, indicating that, in the high-stress range (*σ*_m_ > 611.20 kPa), the cushioning coefficient *C* of foam aluminum remains at a low level, with stable energy absorption efficiency that does not deteriorate rapidly as stress increases. The coordinates corresponding to the lowest point of the *C* − *σm* curve for honeycomb paperboard are (*σ*_m_ = 125.65 kPa, *C* = 2.34), making it the material with the lowest maximum stress corresponding to the optimal cushioning efficiency among the three materials. After the minimum point, the *C* − *σm* curve for honeycomb paperboard rises rapidly, indicating that, in the medium-to-high-stress range (*σ*_m_ > 125.65 kPa), the cushioning coefficient *C* of the honeycomb paperboard increases sharply, and the energy absorption efficiency deteriorates rapidly. The coordinates corresponding to the lowest point on the *C* − *σm* curve for polyurethane foam are (*σ*_m_ = 399.40 kPa, *C* = 2.51). Its cushioning efficiency is stable, and its performance lies between that of aluminum foam and honeycomb paperboard.

Combining the predicted cushioning characteristics, the simulation results suggest that the three materials could potentially form a complementary selection system covering a certain load range. The optimal cushioning efficiency ranges predicted by the model for the three materials correspond to the optimal static stress ranges of the *σ*_s_ − *G*_m_ curves discussed earlier: the simulations indicate that foam aluminum is suitable for high *σ*_s_ (heavy loads), polyurethane for medium *σ*_s_ (medium loads), and honeycomb cardboard for low *σ*_s_ (light loads). These findings support the rationality of the proposed selection criteria from an energy-efficiency perspective within the simulation framework.

### 4.5. Analysis of Airdrop Application Cases

#### 4.5.1. Design Workflow Based on C−σm Curves

**Figure 17:**
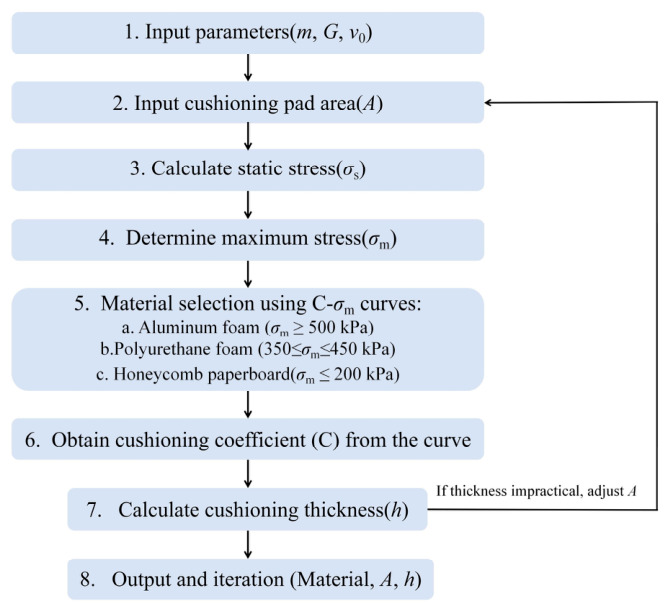
Flowchart of the *C* − *σm* curve-based cushioning design procedure.

Figure 17 shows the design process for cushion dimensions based on the *C* − *σm* curve. First, basic input parameters are collected, including the cargo mass m, the maximum allowable acceleration G (indicating cargo fragility), the impact velocity v0, and the cushion area A. Then, the static stress *σ*_s_ and the maximum impact stress σm acting on the cushion are calculated using the formula. The optimal stress ranges for the three cushioning materials, obtained from the *C* − *σm* curve (Figure 16), are used to select the material by matching the calculated *σ*_m_ with the effective working range. The corresponding cushioning coefficient *C* is determined by drawing a perpendicular line at the target *σ*_m_ and intersecting it with the *C* − *σm* curve, while the required cushioning layer thickness is calculated using Equation (9). This workflow outputs the selected cushioning material and its load-bearing capacity.

#### 4.5.2. Application Cases

Assuming the airdropping of different types of items, the fragility value for precision electronic instruments is generally within 20 g; for aviation instruments, televisions, and similar items, it is around 50 g; for radio equipment, it is around 80 g; and for general machinery, components, and grain, it is above 120 g [28]. Based on this, three scenarios were selected: Case 1 involves 500 kg of mechanical parts with a fragility value of 120 g; Case 2 involves 300 kg of instruments and gauges with a fragility value of 50 g; Case 3 involves 300 kg of precision components with a fragility value of 20 g. The base area of the airdropped item is 1.21 m^2^.

**Figure 18:**
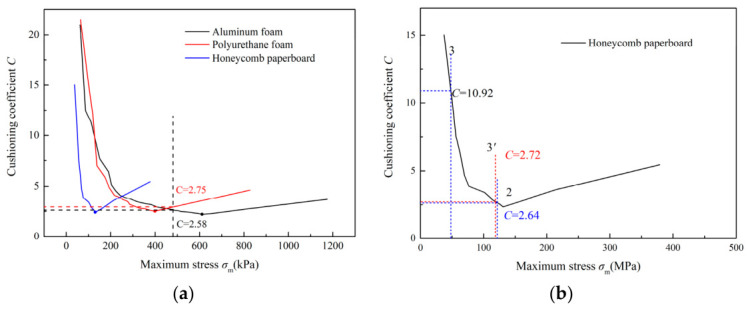
The vertical lines of *σ*_m_ in different cases: (**a**) Case 1; (**b**) Case 2 and 3.

Case 1: The landing velocity of the airdropped item is 6 m/s (corresponding to a drop height of 1.84 m), and full-surface cushioning is employed. Calculations yield *σ*_s_ = 4.05 kPa and *σ*_m_ = 486.45 kPa. Figure 18a shows a vertical line 1 drawn at *σ* = 486.45 kPa on the *x*-axis of the *C* − *σm* curve; the corresponding cushioning coefficients (*C*) for foam aluminum, polyurethane foam, and honeycomb paperboard are 2.59, 2.94, and 6.62, respectively. Based on the value of *σ*_m_, either foam aluminum or polyurethane foam should be selected. Calculating the thickness *h* of the foam aluminum or polyurethane foam cushion using Equation (9) yields 4.0 cm and 4.5 cm, respectively; the difference between the two is negligible, so the choice can be made based on the cost of the two materials. If the landing speed of the airdropped item is increased to 8 m/s (corresponding to a drop height of 3.26 m), the calculated values are *σ*_s_ = 4.05 kPa and *σ*_m_ = 486.45 kPa, which remain unchanged. However, according to Equation (9), the thickness h of the aluminum foam or polyurethane foam cushioning pads becomes 7.0 cm and 8.0 cm, respectively. The increase in drop velocity (corresponding to the drop height) does not affect the static stress *σ*_s_ or the maximum stress *σ*_m_.

Case 2: Using full cushioning, the calculations yield *σ*_s_ = 2.43 kPa and *σ*_m_ = 121.6 kPa. Since the maximum stress is close to the optimal cushioning region of the honeycomb paperboard cushioning curve, as shown by vertical line 2 in Figure 18b, it is selected as the cushioning material. The cushioning coefficient *C* is calculated to be 2.64, and, using Equation (9), *h* is calculated to be 9.7 cm, yielding the minimum thickness *h* = 9.7 cm.

Case 3: Using full cushioning, the calculations yield *σ*_s_ = 2.43 kPa and *σ*_m_ = 48.6 kPa. A vertical line is drawn as shown in Figure 18b (Line 3). Since the vertical line is far from the lowest point of the *C* − *σm* curve, the cushioning coefficient *C* = 10.92 is obtained, and the cushion thickness h = 100.50 cm. At this point, h is too large; if the cushion area is reduced to 0.5 m^2^, the calculations yield *σ*_s_ = 5.88 kPa and *σ*_m_ = 117.7 kPa. As shown by line 3′ in Figure 18b, the cushioning coefficient *C* = 2.72 and the cushion thickness h = 25.0 cm are obtained, with this cushion area and thickness being more appropriate. By adjusting the contact area of the cushion pad, the working stress of the material is increased from 48.6 kPa to 117.7 kPa, bringing it into the optimal cushioning efficiency range. The cushioning coefficient decreases from 10.92 to 2.72, and the cushion pad thickness is significantly reduced from 100.5 cm to 25.0 cm, while strictly meeting the 20g brittle value constraint. This case study demonstrates that, for precision devices with high impact resistance requirements, the working stress can be optimized by adjusting the cushion area, enabling the material to operate within the optimal cushioning efficiency range and achieving the best balance between cushioning performance and structural dimensions.

To verify the accuracy of the design method described above, simulation analyses were conducted using the same drop simulation model as described earlier for the optimal design solutions of the three cases. Identical material parameters, thickness, contact area, and drop velocity were set. The peak acceleration of the dropped component was extracted and compared with the design brittle value to calculate the relative error, as shown in Table 9. The relative errors for all three cases were within 10%, and the simulated peak accelerations were all lower than the design brittle values, thereby meeting the cushioning protection requirements.

## 5. Conclusions

This study simulates the cushioning performance of honeycomb paperboard, polyurethane foam, and aluminum foam using ANSYS/LS-DYNA R15.0. The numerical model was validated against experimental drop impact tests, yielding *C* − *σm* curves for reliable medium-sized airdrop cushioning design. Although the qualitative correspondence between load level and material stiffness may appear intuitive, the quantitative stress boundaries, the co-optimization design workflow, and the stress-tuning principle demonstrated in this study are non-obvious findings that required systematic simulation and experimental validation. The results indicate the following:

(1) All three materials exhibit concave *C* − *σm* curves with a distinct optimal efficiency point. Aluminum foam suits high-stress ranges with stable efficiency and high load capacity; polyurethane foam fits medium loads with balanced performance; honeycomb paperboard offers lightweight, low-cost benefits for low-stress applications. Together, these three materials cover most of the payload range for medium-sized airdrops and form a systematic framework for material selection.
(2) The optimal static stress increases positively with pad thickness, whereas a reduction in thickness lowers the threshold. Aluminum foam’s optimal stress increases 133% with thickness, showing the greatest adaptability gain. Minimum peak acceleration decreases with thickness. Before saturation, maximum stress and energy density in low-stress phases follow a unified constitutive relation (R^2^ > 0.96), enabling accurate prediction of the cushioning performance for any drop height-to-cushion thickness ratio within this range.
(3) A quantitative design method based on the *C* − σm curve has been developed. By adjusting the bearing area, the maximum stress can be shifted to the range of optimal cushioning efficiency, demonstrating that cushioning volume can be significantly reduced while still meeting the protection requirements for fragile items. This principle is not dependent on specific materials and can be applied to other cushioning materials and loading conditions.

Overall, this work contributes (i) a *C* − σ_m_ curve-based cushioning design framework tailored to medium-sized airdrops; (ii) a systematic comparative characterization of three representative material classes under identical airdrop-relevant conditions; and (iii) a material-agnostic stress-tuning principle for co-optimizing cushion thickness and bearing area.

## Figures and Tables

**Table 1:** Physical parameters of materials.

Unit	Materials	Density/kg/m^3^	Elasticity Modulus/MPa	Poisson’s Ratio
Airdrop box	ABS Plastic	1180	2000	0.39
Airdrop platform	Nylon 66	1140	1480	0.41
Bandage	Polyamide	160	1260	0.06
Ground	Concrete	2300	30,000	0.18
Cushioning pad	Polyurethane foam [22]	230	150	0.10
Cushioning pad	Aluminum foam [23]	475	450	0.37

**Table 2:** Mechanical property parameters of honeycomb paperboard core layer and face layer.

Unit	Density/kg/m^3^	Elastic Modulus/MPa	Poisson’s Ratio	Tangential Modulus/kPa	Failure Strain
Sheet layer	700	12,000	0.38	1.2	0.4
Core layer	120	2000	0.38	12.3	0.9

**Table 3:** Data points for the stress–strain curves of polyurethane foam and aluminum foam.

Materials	Type	Value														
Polyurethane Foam [22]	Strain	0	0.02	0.06	0.08	0.12	0.2	0.25	0.3	0.39	0.45	0.51	0.56	0.6	0.62	0.65
	Stress	0	1.98	5.57	6.23	5.72	5.5	5.72	5.65	6.23	6.86	7.99	9.64	12.10	13.97	16.00
Aluminum Foam [23]	Strain	0	0.01	0.02	0.06	0.09	0.13	0.19	0.24	0.29	0.36	0.43	0.51	0.57	0.66	0.75
	Stress	0	3.14	6.65	3.70	4.99	6.10	8.68	9.61	10.16	10.90	12.75	15.89	20.69	33.36	72.32

**Table 4:** Comparison of peak acceleration for grids with different densities.

Grid Reference Size	Peak Acceleration/g
0.75	40.68
1	41.30
1.5	44.35

**Table 5:** Test and simulation of peak acceleration of different weights.

Mass/kg	Peak Acceleration/g		Relative Error/%
	Test	Simulation	
300	52.8	54.6	3.4
500	44.6	41.3	7.4
700	54.4	48.8	10.3

**Table 6:** Simulation and calculation results.

Simulation Data			Calculation Data	
Mass/kg	Static Stress/kPa	Peak Acceleration/g	Maximum Stress/kPa	Cushioning Coefficient
25	0.20	184.16	37.33	15.03
50	0.41	124.06	50.29	10.13
75	0.61	92.233	56.15	7.53
100	0.81	77.23	62.62	6.30
150	1.22	57.22	69.60	4.67
200	1.62	47.35	76.95	3.86
300	2.43	41.69	101.41	3.40
400	3.24	35.14	113.97	2.87
500	4.05	31.25	126.68	2.55
540	4.46	28.75	125.65	2.34
580	4.62	33.11	155.65	2.70
600	4.86	44.20	215.01	3.60
700	5.67	66.68	378.42	5.44

**Table 7:** Optimal static stress of different thicknesses.

Materials Type	Optimal Static Stress of *h* = 50 mm/kPa	Optimal Static Stress of *h* = 150 mm/kPa	Incremental
Aluminum foam	9.72	22.68	133%
Polyurethane foam	6.48	12.96	100%
Honeycomb paperboard	3.24	4.37	35%

**Table 8:** Comparison of the cushioning characteristics of three materials.

Materials Type	Optimal Maximum Stress/kPa	*C*_min_	Stress Range/kPa	Airdrop Payloads	Core Strengths
Aluminum foam	611.2	2.21	>500	Heavy loads	Stable efficiency under high loads and high stresses
Polyurethane foam	399.4	2.51	350~450	Middle loads	Well-balanced performance, suitable for transitional applications
Honeycomb paperboard	125.7	2.34	<200	Light loads	Lightweight, low-cost, and highly efficient under light loads

**Table 9:** Comparison of design results and simulation results for the three case types.

Case Numbers	Item Type	Mass/kg	Fragility Value G/g	Peak Acceleration/g	Error(%)
1	Machine Parts	500	120	110.3	8.08%
2	Instruments and Meters	300	50	45.7	2.60%
3	Precision Components	300	20	19.2	4.00%

## Data Availability

The original contributions presented in this study are included in the article. Further inquiries can be directed to the corresponding authors.

## Supplementary Materials

The following supporting information can be downloaded at: https://www.mdpi.com/article/10.3390/ma19122526/s1, Table S1: Energy density—maximum stress fitted curves data of honeycomb paperboard. Table S2: Energy density—maximum stress fitted curves data of Polyurethane foam. Table S3: Energy density—maximum stress fitted curves data of Aluminum foam. Table S4: *C* − σm curve data of honeycomb paperboard. Table S5: *C* − σm curve data of Polyurethane foam. Table S6: *C* − σm curve data of Aluminum foam.

## Abbreviations

The following abbreviations are used in this manuscript:

*C*	Cushioning coefficient
G	Fragility value
*G_m_*	Peak acceleration
*σ*_m_	Maximum stress
*H*	Fall height
*A*	Bearing area of the cushioning pad
*h*	Thickness of the cushioning pad
*u*	Elastic potential energy of the cushioning pad
