# Supplementary material for: Dynamic Impact Characteristics of Airdrop Cushioning Materials and a C − σm Curve-Based Cushioning Pad Design Method
Source: Materials (Basel). 2026 Jun 11;19(12):2526. doi: 10.3390/ma19122526 (PMC13304024; doi:10.3390/ma19122526)
Supplement: Supplementary file 1 [file materials-19-02526-s001.zip › materials-4343200-supplementary.pdf]

Supplementary Material

Table S1 Energy density - maximum stress fitted curves data of honeycomb paperboard

h=50mm		*h*=100mm		*h*=150mm	
Maximum stress (kPa)	Energy density(kPa)	Maximum stress (kPa)	Energy density(kPa)	Maximum stress (kPa)	Energy density(kPa)
66.841	7.459	48.746	3.729	37.32731	2.486
92.541	14.918	63.252	7.459	50.29093	4.973
110.61	22.377	74.733	11.188	56.14607	7.459
120.992	29.835	83.851	14.918	62.61755	9.945
139.14	44.753	97.012	22.377	69.5916	14.918
153.587	59.671	102.893	29.835	76.78902	19.89
186.555	89.506	128.353	44.753	101.40916	29.835
		154.314	59.671	113.96742	39.78
		162.636	67.13	125.66529	49.726
				130.52975	55.693
				140.77174	57.682

Table S2 Energy density - maximum stress fitted curves data of Polyurethane foam

h=50mm		*h*=100mm		*h*=150mm	
Maximum stress (kPa)	Energy density(kPa)	Maximum stress (kPa)	Energy density(kPa)	Maximum stress (kPa)	Energy density(kPa)
115.560	10.851	93.848	5.475	64.066	3.614
165.090	25.285	121.106	12.642	102.568	8.412
192.306	36.114	133.570	18	119.755	12.023
236.711	72.106	154.661	36.171	138.589	24.047
259.636	108.221	184.364	54.124	174.805	36.065
264.586	117.611	219.491	72.163	193.380	48.091
276.810	126.242	239.665	90.201	217.025	60.112
308.040	144.313	270.679	108.261	241.983	72.134
349.215	180.375	296.337	126.22	254.907	84.151
383.901	216.485	316.575	144.331	276.303	96.174
421.145	252.445	344.566	162.355	285.362	108.192
		370.967	180.369	302.666	120.211
		384.227	198.435	317.089	132.235
		406.245	216.442	333.042	144.264
		431.181	234.412	355.114	156.286
				368.245	168.35
				382.629	180.321
				399.396	192.341
				438.562	204.366

Table S3 Energy density - maximum stress fitted curves data of Aluminum foam

h=50mm		*h*=100mm		*h*=150mm	
Maximum stress (kPa)	Energy density(kPa)	Maximum stress (kPa)	Energy density(kPa)	Maximum stress (kPa)	Energy density(kPa)
114.917	8.951	74.705	4.475	62.521	2.984
170.679	20.885	116.408	10.442	86.615	6.962
194.942	29.835	135.843	14.918	112.000	9.945
244.661	59.671	168.430	29.835	151.901	19.890
284.331	89.506	202.066	44.753	190.636	29.835
337.388	119.341	230.043	59.671	203.815	39.780
375.769	149.177	266.322	74.588	224.008	49.726
427.071	179.012	300.149	89.506	243.650	59.671
470.406	208.848	328.479	104.424	268.117	69.616
505.256	238.683	352.793	119.341	293.302	79.561
518.534	268.518	380.559	134.259	312.932	89.506
552.413	298.354	404.711	149.177	334.124	99.451
599.673	328.189	422.727	164.095	356.455	109.396
		456.188	179.012	379.537	119.341
		477.455	193.930	394.512	129.287
		500.182	208.848	406.926	139.232
		520.326	223.765	425.826	149.177
		535.273	238.683	443.240	159.122
		553.905	253.601	459.983	169.067
		578.990	268.518	476.628	179.012
		605.817	283.436	487.562	188.957
				505.388	198.902
				517.190	208.848
				533.855	218.793
				550.802	228.738
				564.317	238.683
				577.603	248.628
				591.145	258.573
				602.479	268.518

Table S4 C-σ_m curve data of honeycomb paperboard

Maximum stress (kPa)	Cushioning coefficient(*C*)
37.33	15.03
50.29	10.13
56.15	7.53
62.62	6.30
69.60	4.67
76.95	3.86
101.41	3.40
113.97	2.87
126.68	2.55
125.65	2.34
155.65	2.70
215.01	3.60
378.42	5.44

Table S5 C-σ_m curve data of Polyurethane foam

Maximum stress (kPa)	Cushioning coefficient(*C*)
64.066	21.499
102.568	14.751
119.755	12.056
138.589	6.976
174.805	5.866
193.38	4.867
217.025	4.1
241.983	3.886
254.907	3.666
276.303	3.477
285.362	3.192
302.666	3.047
317.089	2.902
333.042	2.794
355.114	2.75
368.245	2.648
382.629	2.568
399.396	2.513
562.321	3.33
827.301	4.627

Table S6 C-σ_m curve data of Aluminum foam

Maximum stress (kPa)	Cushioning coefficient(*C*)
243.65	4.088
268.117	3.856
293.302	3.691
312.932	3.5
334.124	3.364
356.455	3.262
379.537	3.184
394.512	3.055
406.926	2.926
425.826	2.858
443.24	2.789
459.983	2.724
476.628	2.666
487.562	2.583
505.388	2.544
517.19	2.479
533.855	2.443
550.802	2.411
564.317	2.367
577.603	2.326
591.145	2.289
602.479	2.246
611.21	2.198
658.827	2.287
799.835	2.684
1174.744	3.696
